# Conservation of writhe helicity under anti-parallel reconnection

**DOI:** 10.1038/srep09224

**Published:** 2015-03-30

**Authors:** Christian E. Laing, Renzo L. Ricca, De Witt L. Sumners

**Affiliations:** 1 Sequenom Inc., 3595 John Hopkins Court, San Diego, CA 92121, USA; 2 Department of Mathematics & Applications, U. Milano-Bicocca Via Cozzi 55, 20125 Milano, Italy; 3 Department of Mathematics, Florida State University 1017 Academic Way, Tallahassee, FL 32306-4510, USA

## Abstract

Reconnection is a fundamental event in many areas of science, from the interaction of vortices in classical and quantum fluids, and magnetic flux tubes in magnetohydrodynamics and plasma physics, to the recombination in polymer physics and DNA biology. By using fundamental results in topological fluid mechanics, the helicity of a flux tube can be calculated in terms of writhe and twist contributions. Here we show that the writhe is conserved under anti-parallel reconnection. Hence, for a pair of interacting flux tubes of equal flux, if the twist of the reconnected tube is the sum of the original twists of the interacting tubes, then helicity is conserved during reconnection. Thus, any deviation from helicity conservation is entirely due to the intrinsic twist inserted or deleted locally at the reconnection site. This result has important implications for helicity and energy considerations in various physical contexts.

Filamentary structures, such as vortex filaments in classical and quantum fluids^1^,^2^,^3^,^4^, magnetic flux tubes^5^,^6^, phase defects^7^, and polymers and macromolecules^8^,^9^ are ubiquitous in nature. When parts of these filaments come sufficiently close to one another, they tend to influence each other and recombine through reconnections (see Figure 1). Reconnection is a process associated with a change of topology and geometry of the interacting filaments by an exchange of the neighboring strands^10^. In general, when two disjoint, closed tubes (like vortex rings) reconnect, the result is a single closed tube and when a single closed tube reconnects with itself, the result is two closed tubes. Such a topological change is typically accompanied by a change in energy, partly dissipated due to small-scale effects associated with viscosity, resistivity or other. Thus, detailed study of reconnections is crucial to understand energy re-distribution and dissipation in many fluid systems, from vortex tangles in classical and superfluid turbulence^11^,^12^, to phase transitions in mesoscopic physics^7^, from astrophysical flows in solar and stellar physics^6^,^13^ to confined plasmas in fusion physics^14^,^15^. Detailed analysis based on direct numerical simulations of real fluid equations reveals certain qualitative common features of the reconnection event (compare for instance the various scenarios shown in Figure 1). In the majority of cases at the time of closest approach the interacting tubes tend to align themselves in an anti-parallel fashion, followed by a reconnection of the local strands through a rapid, merging process in a direction orthogonal to their mutual alignment before final separation. Fine details of the reconnection event (such as the generation of secondary, bridge structures in vortex dynamics) may differ from case to case, but certain geometric features such as anti-parallel alignment of the reconnecting strands and transversal merging seem to have a generic character. Qualitatively similar features, for instance, seem to characterize recombination events in polymer physics as well as in DNA biology^8^,^16^, when two unknotted circular DNA plasmids are joined into a single plasmid in a site-specific recombination event^9^,^18^,^19^,^17^. These common geometric features are the focus of this paper.

## Results

### Helicity, linking numbers and writhe

**Figure 1:**
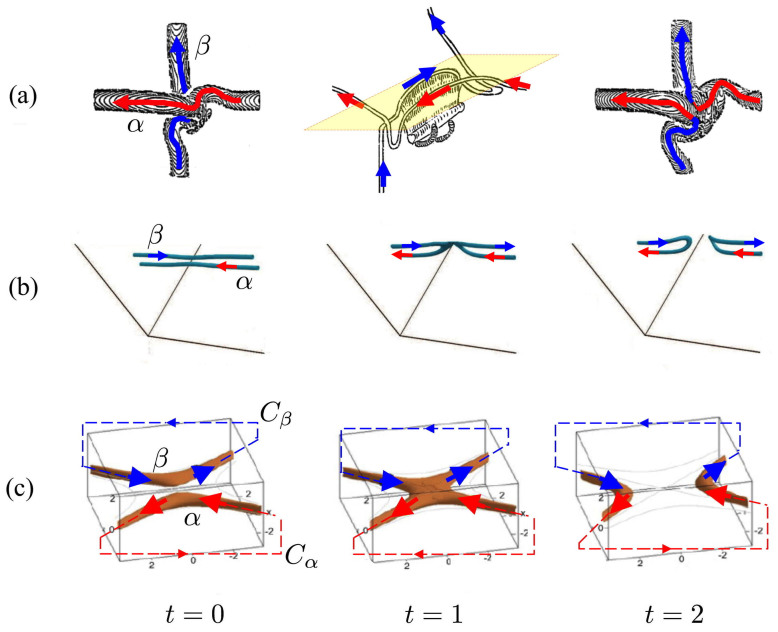
Direct numerical simulations of reconnection events: (a) vortex tubes in a viscous fluid; (b) quantized vortex tubes in superfluid helium; (c) magnetic flux tubes in magnetohydrodynamics. *t* = 0 interaction, *t* = 1 reconnection, *t* = 2 separation of two tube strands *α* and *β* in a real fluid (not visible). (a) Initially orthogonally-offset vortex tubes in a viscous fluid, (b) quantized vortex tubes in superfluid helium, (c) magnetic flux tubes (centered on the spatial curves *C_α_* and *C_β_*) in magnetohydrodynamics. The top, central diagram shows a sketch at the reconnection site (yellow plane), where the vortex strands become locally aligned in an anti-parallel fashion just before reconnection. Images adapted from Refs. 40, 4 and 41, respectively.

In fluid systems a fundamental quantity, that detects topological information and that has a close relation with energy, is the *helicity H* of fluid flows (kinetic or magnetic). For two interacting disjoint tubular filaments *α* and *β*, centered on their respective curves *C_α_* and *C_β_* (see Figure 1c), the helicity *H* = *H*(*α*, *β*) can be written as^20^,^21^,^22^,^23^
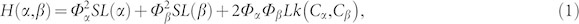
where *Φ* is a measure of the tube flux (field strength), and *SL* and *Lk* are topological numbers denoting self-linking and mutual linking of the two flux-tubes, respectively (for their definitions see Refs. 24, 25, 26, and text below). During reconnection, the interacting tubes may change strength, whereas topology certainly changes; hence a change in helicity should be expected. Even when the flux remains conserved (as in the case of quantized vortices in superfluid helium), a change in linking numbers may happen, because the reconnection of a pair of closed, oriented curves produces a single closed, oriented curve (with no linking number), and vice versa. Here all curves are tacitly assumed to be smooth, with the exception of the polygonal curves referred in the text below and in the next subsection. Polygonal curves are used to facilitate the proof of conservation of writhe under reconnection (since polygonal curves can approximate smooth curves arbitrarily closely). Moreover, we implicitly assume that our smooth curves have nonvanishing curvature almost everywhere (not a very restrictive assumption, since one can always deform a curve with inflexion points in isolation to an inflexion-free curve by a *C*^2^ infinitesimally small perturbation of the original curve, without any appreciable effect on energy).

Since reconnection is a local process, the morphological and structural change experienced by the reconnecting strands is reflected in the change of the individual self-linking numbers. For a single flux tube *α*, *SL*(*α*) admits decomposition into two geometric quantities, the writhe *Wr*(*C_α_*) of the tube centerline *C_α_* and the twist *Tw*(*R_α_*) of the tube reference ribbon *R_α_*^27^; from standard differential geometry, the twist can be decomposed into two parts, given by the normalized total torsion *T*(*C_α_*) of *C_α_*, and the intrinsic twist *N*(*R_α_*) of *R_α_* around *C_α_*. Thus, we have
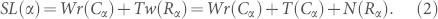
Since writhe and twist are geometric quantities, their values change continuously with the continuous change in space of the curve *C_α_* and the reference ribbon *R_α_*.

Writhe is a geometric measure of *non-planarity* for spatial curves^27^,^28^; indeed, planar curves and closed curves on a round 2-sphere have *zero* writhe. Let the unit sphere *S*^2^ denote the space of directions (unit vectors) in 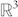. Given an oriented, simple, closed curve *A* in 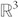, consider a generic planar projection (knot diagram) of *A* in the direction *ν* ∈ *S*^2^, with standard sign convention of ±1 for over/under–passes. One now adds up all of the signed crossings to obtain the *directional writhe* of *A*, *ω_ν_*(*A*). By averaging the directional writhe over all directions, i.e. by summing algebraically the contributions *ω_ν_*(*A*) given by all possible projection directions *ν* ∈ *S*^2^, one obtains the *writhe* of *A*:
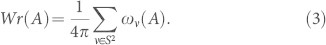
Given a pair of disjoint, simple, closed curves {*A*, *B*}, the *linking number Lk*(*A*, *B*) can be calculated from any generic projection of the pair of curves by adding up the crossings between the curves (neglect the self-crossings of each curve) as follows. Suppose that there are *n* crossings {*X_i_*, 1 ≤ *i* ≤ *n*} between *A* and *B*, and 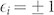 denotes the sign of the *i*-th crossing according as the crossing is positive or negative, then we have
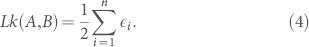
Since the linking number is constant over all projections, averaging the value over all projections does not change this value.

Suppose now that *A* is an oriented *n*-edge polygon with edges {*a_i_*, 1 ≤ *i* ≤ *n*}, and *B* is an *m*-edge polygon with edges {*b_j_*, 1 ≤ *j* ≤ *m*}. Consider a pair of distinct oriented edges {*a_i_*, *a_j_*} of *A*. Following Banchoff^29^ we wish to compute the contribution to the writhe of *A* from the pair of edges {*a_i_*, *a_j_*}. The set of all directions on *S*^2^, where one sees a single crossing between these edges, is an open set; moreover, one sees the same crossing sign over this entire open set. Under the antipodal map on *S*^2^, a map that takes any point *x* ∈ *S*^2^ to −*x*, this open set is invariant, since a crossing seen in a given direction is seen as a crossing of the same sign in the opposite direction. The contribution to the writhe of *A* from the pair of edges {*a_i_*, *a_j_*} is *ω*(*a_i_*, *a_j_*), the signed area on the unit 2-sphere *S*^2^ of this open set. Note that *ω*(*a_i_*, *a_j_*) = 0 if *i* = *j*, or if the edges meet in a common vertex — in each case the edges are identical or co-planar, with no crossings visible under any projection direction. We can compute *Wr*(*A*) in terms of the edges of polygon *A*:
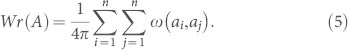
For disjoint oriented polygons {*A*, *B*}, we can compute *Lk*(*A*, *B*) in terms of the edges
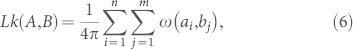
and similarly the writhe of the disjoint union of *A* and *B*:
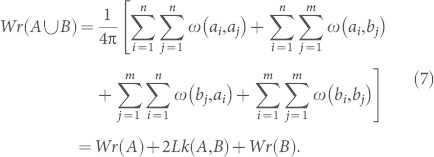


### Reconnection conserves writhe

**Figure 2:**
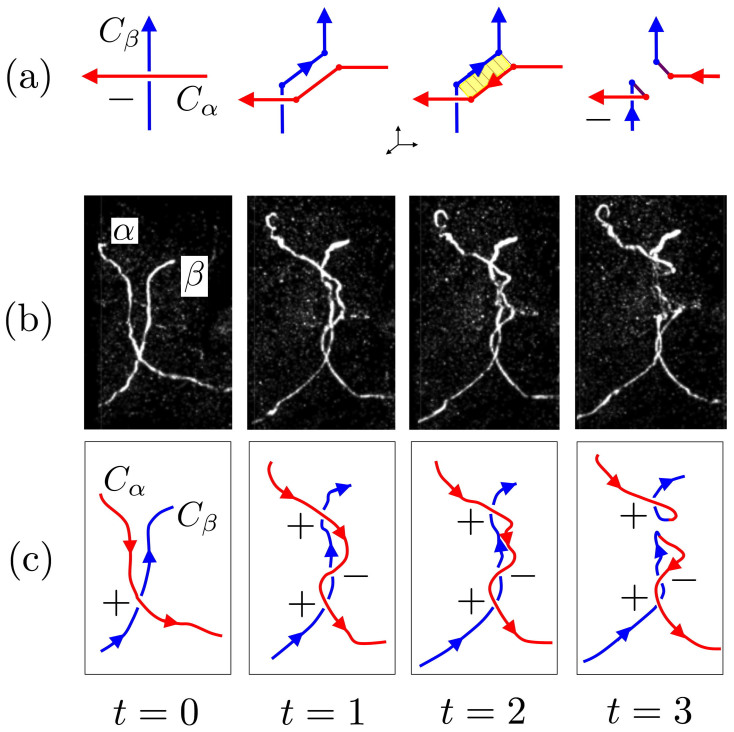
(a) Diagrammatic reconnection of polygonal curves near a crossing; (b) screen shots of a vortex reconnection; (c) smooth tracings of screen shots. (a) Reconnection of two oriented (polygonal) curves near a crossing does not change the writhe (since inscribed polygonal curves can approximate smooth curves arbitrarily closely, in this example we use polygonal curves). We assume that the curves remains almost co-planar at the crossing site, hence in all cases *Wr* ≈ −1. Note the production of the ‘pigtail’ (fourth diagram), due to the mutual cancellation of the anti-parallel strands (yellow region in third diagram). (b) Screen shots of the anti-parallel alignment and subsequent reconnection of two strands of a trefoil vortex knot from the experiment of Kleckner and Irvine^30^ (reproduced with permission). (c) The apparent crossings at each time sequence *t* = 0, 1, 2, 3 (red curve over blue curve) are the original overpasses of the same strands above. The stage just after reconnection is shown in *t* = 3. The directional writhe in each of the figures at *t* = 0, 1, 2, 3 is +1. Compare this scenario with the idealized sketches above.

Experimental and computational evidence shows that reconnection is a process that takes place along the interacting segments of two tube centerlines (see Figure 2b), and does not occur at a point in isolation. Hence, when the interacting segments of two tubes approach each other, the reconnection event can only take place *near* an apparent crossing point (and not *at* a crossing point, that in any case depends on the projection direction). Directional writhe, on the other hand, depends on the projection, and only when it is averaged over all directions of sight it becomes a projection independent measure (as in eq. (3)). Thus, reconnection near a crossing does not change the writhe (see Figure 1a). Figure 2b shows close up screen shots of the anti-parallel alignment of two trefoil vortex strands and subsequent reconnection from the experiment of Kleckner and Irvine^30^. From direct inspection of the supplementary material made available by *Nature Physics*, we can see (from the smooth tracings of Figure 2c) that the red vortex line has been moved across the top of the blue vortex line (*t* = 0, 1) and then the anti-parallel reconnection segments are spatially juxtaposed (*t* = 2). The configuration just after reconnection is shown in *t* = 3. The directional writhe in each of the figures at *t* = 0, 1, 2, 3 is +1. This reconnection event is very fast compared with the typical vortex evolution time, so that the writhe of the unseen rest of the configuration remains essentially constant throughout this quick reconnection. Although we only have one projection direction shown in the screen shots, the pair of vortex segments are very close to co-planar just before and just after reconnection takes place, so the directional writhe is very close to the true writhe. In this experiment, we see that observed reconnection of the trefoil vortex to the Hopf link vortex conserves writhe.

A rigorous proof that anti-parallel reconnection conserves writhe is given here below. Our result will not depend on any specific projection and proof relies on the following assumptions:

A1: under reconnection, orientation is preserved;

A2: the reconnecting segments are oriented in an anti-parallel fashion;

A3: the reconnecting segments are *isomorphic*, identical under spatial translation.

**Figure 3:**
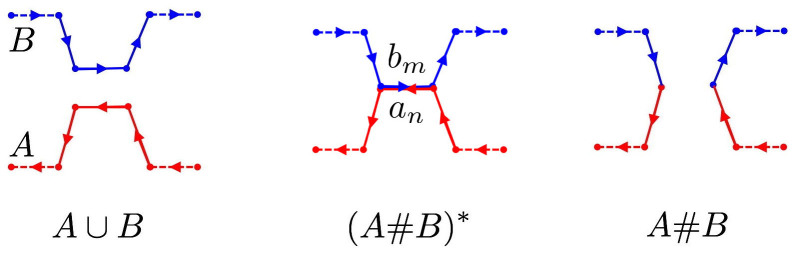
Reconnection of polygonal curves with intermediate *θ*-curve at the center. Reconnection of polygonal curves *A* and *B*: the intermediate *θ*-curve (*A*#*B*)* at the center has two coincident and oppositely oriented edges *a_n_* and *b_m_*.

Now, suppose that we have two disjoint oriented polygons *A* = {*a_i_*, 1 ≤ *i* ≤ *n*} and *B* = {*b_j_*, 1 ≤ *j* ≤ *m*}, that have the following properties:

(i) edges *a_n_* and *b_m_* have the same length;

(ii) polygon *B* can be translated without intersecting polygon *A* until the edges *a_n_* and *b_m_* are coincident with opposite orientation (as in the central diagram of Figure 3).

When edges *a_n_* and *b_m_* are coincident, one has formed the *θ*-curve intermediate (*A*#*B*)*; by deleting the interior of the common edge *a_n_* = *b_m_* from (*A*#*B*)*, one obtains the oriented reconnected curve (*A*#*B*).

Consider the effect of the translation that aligns *b_m_* with *a_n_* on each of the terms in equation (7) for 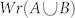: since translation is a rigid motion, *Wr*(*A*) and *Wr*(*B*) are unchanged during the translation, and 2*Lk*(*A*, *B*) is a topological invariant unchanged by translation. At the end of translation, when *a_n_* = *b_m_*, if we stipulate that in the calculation of *Wr*[(*A*#*B*)*] we will count the common edge *a_n_* = *b_m_* twice (with opposite orientations for *a_n_* and *b_m_*), then we have shown
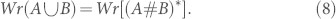
Since *a_n_* = *b_m_* with opposite orientations, for each edge *e* in 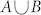, we have *ω*(*a_n_*, *e*) = −*ω*(*b_m_*, *e*), so in the calculation for *Wr*[(*A*#*B*)*] these terms cancel out in pairs, and we are left with the writhe of the reconnected curve (*A*#*B*), and we have proved:

**Theorem 1** *Reconnection conserves writhe: for disjoint oriented polygons A and B (satisfying properties (i) and (ii) above),* 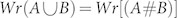.

When a single curve reconnects with itself to produce a pair of curves, the writhe of the single curve may change as the reconnection segments are aligned and brought into spatial juxtaposition. However, as the segments to be juxtaposed are moved closer and closer together, the writhe of the configuration approaches a limiting value, the writhe of the theta-curve intermediate. This limiting value of the writhe is equal to the writhe of the reconnected pair of disjoint curves.

### Conservation of helicity under anti-parallel reconnection

**Figure 4:**
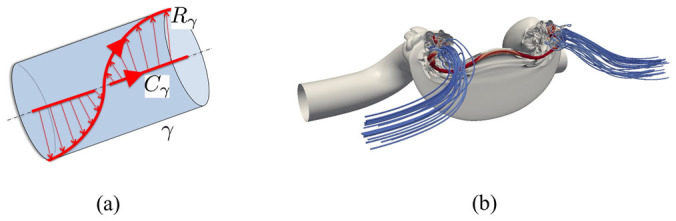
(a) Flux tube *γ* centered on spatial curve *C_γ_*; (b) vortex lines (blue) and vorticity isosurface (solid grey) under reconnection. (a) The ribbon *R_γ_* is formed by connecting *C_γ_* with one of the field lines in *γ*. (b) Note the bridge region (threaded by the red line) formed by the re-organization of the weaker vorticity. From a direct numerical simulation of the Navier-Stokes equations^11^.

Figure 4a shows the flux tube *γ*, with center curve *C_γ_* and flux ribbon *R_γ_*, formed by connecting *C_γ_* with one of the field lines in *γ*. Suppose also that flux tube *γ* has flux *Φ*. For a single flux tube *γ* eqs. (1) and (2) give us
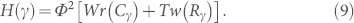
By using the right-hand side decomposition given by eq. (2), we can distinguish the *centerline helicity H_C_* = *Φ*^2^[*Wr*(*C_γ_*) + *T*(*C_γ_*)], that depends solely on tube axis geometry (so that can be entirely estimated by external measurements of *C_γ_*), from the *intrinsic twist helicity H_N_* = *Φ*^2^*N*(*R_γ_*), that depends on the internal twist of the field line distribution. Let *T*(*s*) denote the unit tangent vector at position *s* on the curve *C_γ_* (parameterized by arc length *s*), and *V*(*s*) denote a unit normal vector pointing from *C_γ_* to the edge of ribbon *R_γ_* at position *s*. The *incremental twist* of the ribbon *R_γ_* along the center line *C_γ_* (in the direction of *T*) at position *s* is given by 
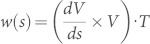
 (see Refs. 22, 27). The *total twist* is thus given by the line integral:
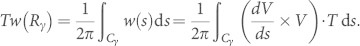
Suppose now that we have two disjoint flux tubes {*α*, *β*} with equal flux *Φ*. Take *Φ* = 1 for simplicity. Suppose also that the oriented center lines of tubes *α* and *β* satisfy the smooth version of conditions (i) and (ii) of Theorem 1 above for reconnection. Specifically, center lines *C_α_* and *C_β_* are each divided into two arcs: 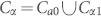, and 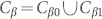. In the reconnection event, *C_β_* is translated (without crossing *C_α_*) until arcs *C_α_*_0_ and *C_β_*_0_ are coincident (with opposite orientation), producing the *θ*-curve intermediate (*C_α_*#*C_β_*)*. At this time, the (infinitesimally small) coincident arc *C_α_*_0_ = *C_β_*_0_ is removed, producing the reconnected curve 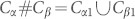. Before reconnection (see, for example, Figure 1c), we have:
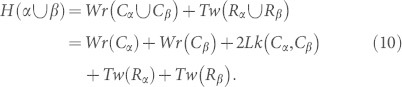
Preliminary results along the lines of the last eq. (10), based on linking numbers and mutual winding of magnetic lines (but not on writhe and twist decomposition), can be found in Ref. 14. Since the ribbons *R_α_* and *R_β_* are disjoint, then the twist of the union of the ribbons is the sum of the individual twists of each ribbon. Given that the flux tubes are locally aligned for reconnection, then translating *C_β_* to *C_α_* conserves the individual twist integrals.

For the *θ*-curve intermediate, we assume that the superimposed arc 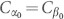 has both ribbons on it, so the twist of this ribbon over the *θ*-curve intermediate (*C_α_*#*C_β_*)* has total twist the sum of the individual twists. The twist of the ribbon over the reconnected center lines *R*(*C_α_*#*C_β_*) is 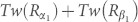. We have the following equation for the change in twist due to reconnection:
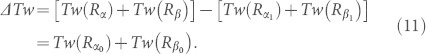
In a reconnection event suppose now that twist is conserved, i.e.
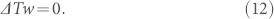
Given this, we have conservation of helicity:

**Theorem 2** *Given anti-parallel reconnection of flux tubes* {*α, β*} *with equal flux Φ, if the total twist of the flux tube ribbons is conserved, then helicity is also conserved, that is*
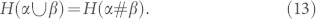


### Role of twist

Since the super-imposed edges have opposite orientation, it is possible that the line integrals over the edges have the same absolute value and different sign, giving us *ΔTw* = 0. Moreover, the edges that get superimposed to form the *θ*-curve intermediate can have vanishingly small length (or take the limit as the length of the super-imposed edge goes to zero). At zero length (the *θ*-curve intermediate now becomes a figure-of-eight, where *C_α_* and *C_β_* have a vertex in common), the line integrals over the common vertex vanish, and *ΔTw* = 0. This may be the case for reconnections of quantized vortex filaments in superfluids, whose typical vortex core cross-section is of the order of 10^−10^ m in Helium–4, several orders of magnitudes smaller than the average distance between vortices in typical laboratory experiments^2^. Furthermore, since a quantized vortex filament is essentially an empty cavity, we have no intrinsic twist, hence total twist reduces to total torsion (cf. eq. 2). Lack of internal structure, and hence of intrinsic twist, characterizes many other physical systems, such as atomic Bose–Einstein condensates^31^, phase line singularities in nonlinear optics^32^ and, possibly, superconductors^33^, where reconnections may indeed trigger topologically complex structures. For all these systems any change in self-linking number (and helicity) should be ascribed to the sole change in total torsion through reconnection.

As mentioned in the introduction (see again eq. 2), suppose that the smooth curve *C_α_* is parameterized by arc-length *s*, and that *τ*(*s*) denotes the torsion at a point on the curve. The normalized total torsion *T*(*C_α_*) of *C_α_* is given by the integral
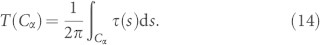
Suppose now that smooth curves *C_α_* and *C_β_* are to be reconnected (in an anti-parallel fashion). The normalized total torsion of the reconnected curve is given by the integral
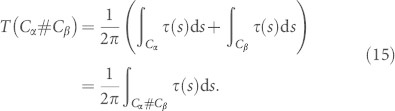
Since for infinitesimally small, anti-parallel, co-planar arcs *T*(*C_α_*_0_) = −*T*(*C_β_*_0_) = 0 (total torsion is additive), we must have 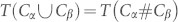. Hence,

**Corollary 1** *If the intrinsic twist N*(*R_α_*_0_) ≠ *N*(*R_β_*_0_)*, then*
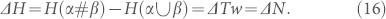
Since total torsion is due to the contribution of the torsion of the tube axes over their entire length, a quantity that can be estimated or computed directly, any change in conformational energy through reconnection can be estimated via total torsion information quite accurately. Note that since reconnection does not take place at a point, inflexion points in isolation are neither assumed to arise nor, if they do, to have any particular effect in the process^22^. When intrinsic twist is an important part of total twist (see Figure 4b), careful considerations on the relative role of spatial gradients associated with curvature and torsion of the tube axis and intrinsic twist must be made. Since dissipative forces tend to erode higher order gradients first, it is natural to expect that, in general, *ΔN* ≠ 0. Hence, as a consequence of Theorem 2 above, any change in helicity should be ascribed to the sole change in intrinsic twist.

## Discussion

We have proven that total writhe remains conserved under anti-parallel reconnection of flux tube strands. Since the helicity of a flux tube admits decomposition in terms of writhe and twist, this result implies that for a pair of interacting flux tubes of equal flux, writhe helicity remains conserved throughout the reconnection process. In this case any deviation from helicity conservation is entirely due to the intrinsic twist inserted or deleted locally at the reconnection site. If the twist of the reconnected tube is the sum of the original twists of the individual tubes before reconnection, then the flux tube helicity is conserved during reconnection.

The analogue of flux tube reconnection in molecular biology is site-specific recombination with directly repeated reconnection sites. The sites are oriented in anti-parallel alignment, and reconnection of a single DNA plasmid produces a pair of plasmids, and reconnection of a pair of plasmids produces a single plasmid. Recent very interesting work on the minimal DNA recombination pathway^34^ proves that if one starts with the trefoil, and insists that recombination reduces configuration complexity (minimal crossing number), then the minimal pathway trefoil → Hopf link → unknotted circle → pair of unknotted, unlinked circles is exactly the reconnection pathway taken by the trefoil vortex in the Kleckner–Irvine experiment^30^.

Our result has therefore important implications well beyond fluid mechanics. For physical systems where helicity and energy considerations are important, and in particular for magnetic fields in solar and plasma physics and for vortex flows in quantum and classical turbulence, reconnections are not only key to understand geometric and topological changes in the fluid flow structure^5^,^30^,^35^,^36^,^37^, but they are also responsible for crucial re-distribution and dissipation of the energy at smaller scales^11^,^12^,^38^,^39^. Our present results will help to address the focus of current research on the role of twist and on the finer details of the tube internal structure undergoing reconnection.

## Author Contributions

The proof of conservation of writhe was done by C.E.L. and D.W.S. The applications to helicity were done by R.L.R. and D.W.S. The paper was written by C.E.L., R.L.R. and D.W.S. Revisions following the suggestions of referees were done by R.L.R.
